# MIRRAGGE – Minimum Information Required for Reproducible AGGregation Experiments

**DOI:** 10.3389/fnmol.2020.582488

**Published:** 2020-11-27

**Authors:** Pedro M. Martins, Susanna Navarro, Alexandra Silva, Maria F. Pinto, Zsuzsa Sárkány, Francisco Figueiredo, Pedro José Barbosa Pereira, Francisca Pinheiro, Zuzana Bednarikova, Michał Burdukiewicz, Oxana V. Galzitskaya, Zuzana Gazova, Cláudio M. Gomes, Annalisa Pastore, Louise C. Serpell, Rostislav Skrabana, Vytautas Smirnovas, Mantas Ziaunys, Daniel E. Otzen, Salvador Ventura, Sandra Macedo-Ribeiro

**Affiliations:** ^1^Instituto de Biologia Molecular e Celular and Instituto de Investigação e Inovação em Saúde, Universidade do Porto, Porto, Portugal; ^2^Instituto de Ciências Biomédicas Abel Salazar, Universidade do Porto, Porto, Portugal; ^3^Institut de Biotecnologia i Biomedicina – Departament de Bioquímica i Biologia Molecular, Universitat Autònoma de Barcelona, Bellaterra, Spain; ^4^International Iberian Nanotechnology Laboratory – Department of Atomic Structure – Composition of Materials, Braga, Portugal; ^5^Department of Biophysics, Institute of Experimental Physics, Slovak Academy of Sciences, Kosice, Slovakia; ^6^Faculty of Mathematics and Information Science, Warsaw University of Technology, Warsaw, Poland; ^7^Institute of Protein Research, Russian Academy of Sciences, Pushchino, Russia; ^8^Institute of Theoretical and Experimental Biophysics, Russian Academy of Sciences, Pushchino, Russia; ^9^Biosystems and Integrative Sciences Institute and Departamento de Química e Bioquímica, Faculdade de Ciências, Universidade de Lisboa, Lisbon, Portugal; ^10^UK-DRI Centre at King’s College London, the Maurice Wohl Clinical Neuroscience Institute, London, United Kingdom; ^11^Sussex Neuroscience, School of Life Sciences, University of Sussex, Brighton, United Kingdom; ^12^Department of Neuroimmunology, Axon Neuroscience R&D Services SE, Bratislava, Slovakia; ^13^Institute of Neuroimmunology, Slovak Academy of Sciences, Bratislava, Slovakia; ^14^Institute of Biotechnology, Life Sciences Center, Vilnius University, Vilnius, Lithuania; ^15^Interdisciplinary Nanoscience Center (iNANO) and Department of Molecular Biology and Genetics, Aarhus University, Aarhus, Denmark

**Keywords:** amyloid, reproducible data, protein, peptide, phase separation

## Abstract

Reports on phase separation and amyloid formation for multiple proteins and aggregation-prone peptides are recurrently used to explore the molecular mechanisms associated with several human diseases. The information conveyed by these reports can be used directly in translational investigation, e.g., for the design of better drug screening strategies, or be compiled in databases for benchmarking novel aggregation-predicting algorithms. Given that minute protocol variations determine different outcomes of protein aggregation assays, there is a strong urge for standardized descriptions of the different types of aggregates and the detailed methods used in their production. In an attempt to address this need, we assembled the Minimum Information Required for Reproducible Aggregation Experiments (MIRRAGGE) guidelines, considering first-principles and the established literature on protein self-assembly and aggregation. This consensus information aims to cover the major and subtle determinants of experimental reproducibility while avoiding excessive technical details that are of limited practical interest for non-specialized users. The MIRRAGGE table (template available in Supplementary Information) is useful as a guide for the design of new studies and as a checklist during submission of experimental reports for publication. Full disclosure of relevant information also enables other researchers to reproduce results correctly and facilitates systematic data deposition into curated databases.

## Introduction

**FIGURE 1:**
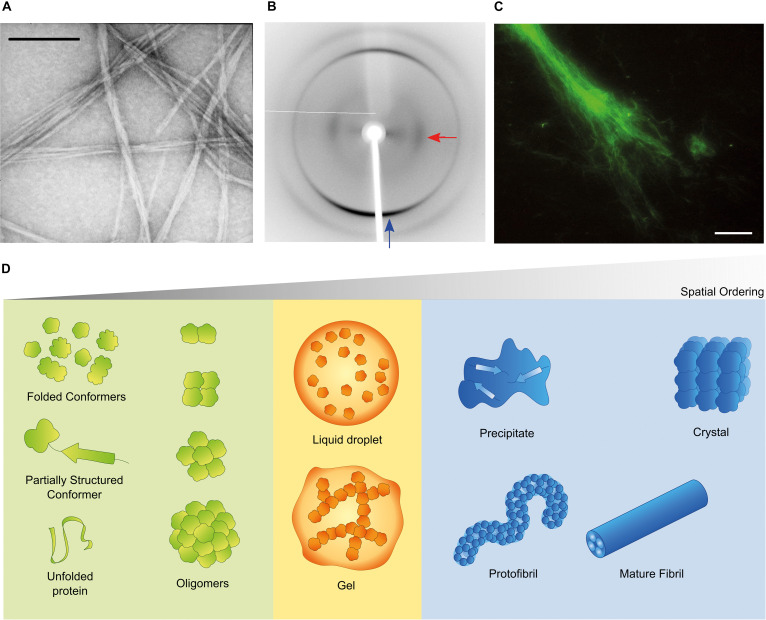
Amyloid and non-amyloid aggregates. **(A)** Amyloids have the characteristic fibrillar appearance illustrated in this transmission electron microscopy (TEM) image of negatively stained fibrils from a peptide derived from the SH3 domain of PI3-kinase (SH3-PI3K) (Ventura et al., 2004). The bar corresponds to 200 nm. **(B)** The ordered β-sheet structure of amyloid fibrils produces the cross-β X-ray diffraction pattern here exemplified for fibrils of a peptide derived from Mot3 yeast prion (Sant’Anna et al., 2016; Fernandez et al., 2017). The blue and red arrows indicate typical reflections at 4.7 and 10.2 Å, respectively. **(C)** Thioflavin-S (Th-S) is an histological dye that shows fluorescence in presence of amyloid fibrils (example of SH3-PI3K fibrils stained with Th-S) (Ventura et al., 2004). The bar corresponds to 20 μm. **(D)** Folded and misfolded monomers are in dynamic equilibrium with soluble oligomers and may coexist in solution with different condensates, such as dense liquids and gels, amyloid fibrils and their protofibrillar precursors, crystals and amorphous solids. For simplicity, the more complex cases of fibril polymorphism, multiprotein assemblies or nucleic acid-protein assemblies are not shown, and amyloid fibrils are represented as composed of four protofilament units. **(B)** Adapted from (Sant’Anna et al., 2016). All copyright permissions have been secured by the authors.

Aggregation of misfolded proteins and peptides is associated with common and rare neurodegenerative disorders and with amyloidoses (Aguzzi and O’Connor, 2010; Chiti and Dobson, 2017; Benson et al., 2018). Classical neuropathological hallmarks such as neurofibrillary tangles in Alzheimer’s disease (AD), Lewy bodies in Parkinson’s disease (PD), or inclusion bodies in Huntington’s disease, have as main constituent characteristic polypeptides aggregated in the form of insoluble highly-ordered structures named amyloid fibrils (Westermark et al., 2007; Gremer et al., 2017). The definition of amyloid encompasses morphological (Figure 1A), structural (Figure 1B) and histological (Figure 1C) aspects. Amyloid polymorphs are associated with different clinical sub-types of the same disease (Guo et al., 2013; Qiang et al., 2017; Goedert et al., 2018), and they exhibit structural differences that can be characterized at atomic resolution using solid-state nuclear magnetic resonance with magic angle spinning (ss-NMR) and cryo-electron microscopy (cryo-EM) techniques (Fitzpatrick et al., 2013; Qiang et al., 2017; Rasmussen et al., 2017; Iadanza et al., 2018b). When the amyloid fold is exploited by nature for functional purposes, the structural variability is less evident than in disease-related aggregates, thus suggesting that functional amyloids are the result of naturally evolved amino acid sequences (Otzen and Riek, 2019). The analogy of a low-energy “black hole” has been used to illustrate the propensity of proteins to form amyloid fibrils after incomplete native folding or if subjected to appropriate denaturing conditions; under mildly denaturing conditions, native interactions and intrachain interactions are in competition, so that the occurrence of transiently stable, non-native conformations may trigger amyloid fibril formation (Zheng et al., 2013). However, there are other energetically stable structures into which proteins self-assemble irrespectively of, prior to, or concomitantly with the formation of amyloid fibrils (Figure 1D). Of these, the soluble aggregates of amyloidogenic proteins attract a particular interest justified by the cytotoxic properties attributed to amyloid-β (Aβ) oligomers in AD (Ahmed et al., 2010; Yang et al., 2017) and α-synuclein oligomers in PD (Lorenzen et al., 2014; Ingelsson, 2016). Liquid-liquid phase separation is now recognized as having a central role in cell physiology and disease (Shin and Brangwynne, 2017). For example, membraneless compartments of concentrated proteins/nucleic acids are implicated in diverse processes, including RNA metabolism, ribosome assembly, DNA repair and intracellular signaling (Banani et al., 2017). When the capacity of protein quality control by the proteasome is exceeded, misfolded proteins are sequestered into intracellular compartments such as the aggresome, a perinuclear deposit destined to autophagy (Johnston et al., 1998), or the CytoQ and INQ, which are deposition sites of misfolded proteins in the cytosol and the nucleus, respectively (Miller et al., 2015). On the other hand, a neuropathological hallmark of sporadic and inherited forms of amyotrophic lateral sclerosis and frontotemporal dementia is the deposition of poorly soluble assemblies of mutated RNA-binding proteins in the nucleus and cytoplasm of neurons (Patel et al., 2015). The pathogenic mutants are characterized by a diminished ability to reiteratively shift between dispersed and condensed phases consisting of dense liquids and gels (Murakami et al., 2015). The physical properties of the different polypeptide assemblies (summarized in Figure 1D) are determined by the degrees of molecular and supramolecular order. Although denser liquid phases relax more slowly in response to shear deformation, they still lack the long-range translational order characteristic of solids (Falahati and Haji-Akbari, 2019). The maximal organization provided by crystal lattices allows the structure of folded proteins to be solved using X-ray crystallography. Naturally occurring microcrystals are also used by living cells for protein storage, protection and stabilization (Schönherr et al., 2018), whereas in crystallopathies such as eosinophilic inflammation, protein crystals have been reported as promising drug targets (Persson et al., 2019).

## Thermodynamic and Kinetic Obstacles to Reproducibility

According to the phase rule of thermodynamics, the maximum number of stable phases that can coexist within a mixture is limited to *k+2*, with *k* being the number of non-reactive components present in the mixture (Falahati and Haji-Akbari, 2019). The reduced number of accessible microstates due to demixing implies an entropic penalty of −*T*Δ*S* > 0 reflecting the disorder-to-order transition (Shin and Brangwynne, 2017; Falahati and Haji-Akbari, 2019). This entropic cost, which is higher at higher temperature (*T*), can be compensated by the enthalpic contribution (Δ*H* < 0) provided that the new intermolecular interactions are sufficiently strong to decrease the Gibbs free energy Δ*G* = ΔH-TΔ*S*. Whether a given protein undergoes a phase separation or not is, therefore, predictable according to the information of temperature and composition given in phase diagrams. In practice, however, differences in the protein source and sample manipulation generate aggregation states that are not readily duplicated among distinct laboratories, a problem that was highlighted in the editorial entitled “State of Aggregation” of Nature Neuroscience in April, 2011. The difficulties in reproducing protein self-assembly experiments are correlated with the occurrence of metastable states of protein folding (Sohl et al., 1998) and phase separation (Gazit, 2002; Baldwin et al., 2011) consisting of local free energy minima whose evolution toward the global minimum takes place over too long timescales to be biologically realistic. Partially unfolded states that are more stable than the native fold (Giri Rao and Gosavi, 2018) and the kinetic trapping of condensates into oligomeric (Miti et al., 2015) and gel-like states (Alberti, 2017) accentuate the need for a clear description of the initial state of the protein, its source and how it was manipulated.

The interplay between protein folding, oligomerization and metastable phase separation adds uncertainty to the dynamic distribution of the different species during test-tube experiments. How cells spatiotemporally regulate these processes is additionally determined by the occurrence of post-translational modifications (Boeynaems et al., 2018; González et al., 2019), chaperone recruitment (Mateju et al., 2017) and macromolecular crowding (Aguzzi and Altmeyer, 2016; Rivas and Minton, 2016). For the molecular-level understanding of protein aggregation, kinetic results obtained under tightly controlled conditions and in the presence of specific dyes, such as Thioflavin-T (Th-T), Thioflavin-S (Th-S) and Congo red (CR), are analyzed using different nucleation-and-growth models (Crespo et al., 2012; Meisl et al., 2016; Chatani and Yamamoto, 2018). A combination of complementary techniques including (but not limited to) chromatography, light scattering, and advanced microscopy is required to validate complex mechanisms that may comprise the formation of intermediate phases (Ianiro et al., 2019), precursor oligomers (Pieri et al., 2016), reversible oligomerization (Silva et al., 2017), irreversible oligomerization (Silva et al., 2018), and conformational transitions (Ruff et al., 2019), to cite but a few examples. As a stochastic process, primary nucleation is itself a source of variability in which stable clusters are formed occasionally as the result of random molecular collisions (Vekilov, 2010; Sleutel and Van Driessche, 2018). When nucleation is a rare event, the emergence of measurable amounts of protein aggregates is preceded by a lag phase whose variable duration reflects the probability distribution of a successful event (Crespo et al., 2012; Michaels et al., 2016). During amyloid fibril formation, prior addition of >1% of preformed fibrils (or seeds) is often sufficient to eliminate the lag phase and the intrinsic uncertainty associated to primary nucleation (Sárkány et al., 2019). Conversely, protein samples containing residual, yet variable, amounts of preformed seeds may not be suitable for “unseeded” assays due to irreproducible lag times (Crespo et al., 2012, 2016). In these cases, a final step of sample polishing immediately before the aggregation assay is recommended to eliminate vestigial assemblies formed during storage or upon thawing (Mahler et al., 2009; Silva et al., 2017; Weinbuch et al., 2018).

## An Ontological Approach to Protein Aggregation

**FIGURE 2:**
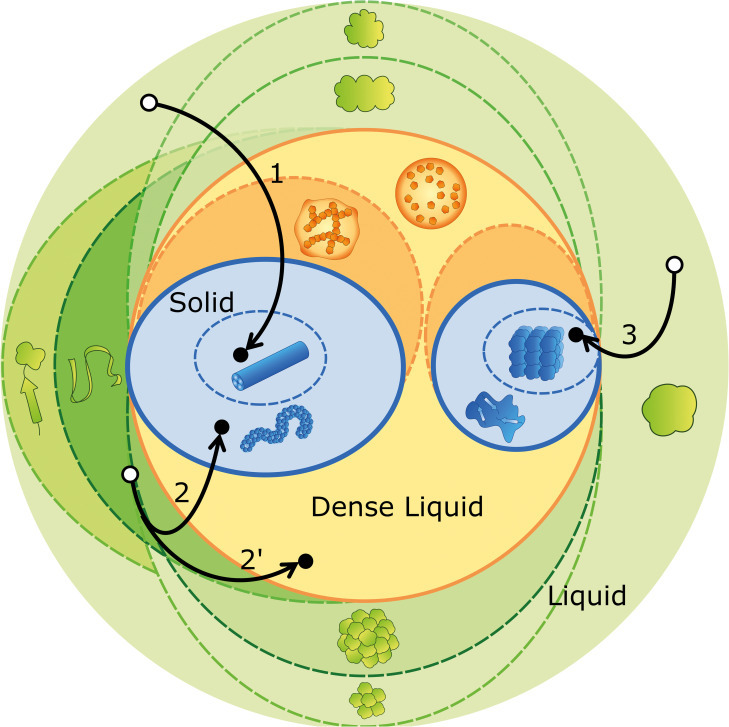
Ontological map of protein aggregation. Phase equilibria (solid outlines) are established between the liquid phase (green circle), the dense liquid phase (orange circle) and the solid phase (blue circles). The different species (represented as in Figure 1D) may undergo structural transitions (dashed lines) that originate distinct species within the same stable phase (different shades of color). Visual examples of aggregation pathways are provided, comprising many (arrow 1), some (arrow 2) and no (arrow 3) intermediate states. Off-pathway aggregation occurring in parallel with amyloid fibrillation is represented by a bifurcation (arrow 2′). The outcome of phase separation (black dots) is contingent on concentration, temperature, pH, ionic strength, amino acid sequence, etc., but also on the initial state of the protein, as it determines the point in the green region from where the experiments depart (white dots).

As new published data on protein aggregation and phase separation proliferate, they increase the risk that different semantics are adopted to characterize the same entities, or else, that different entities end up having the same designation. Motivated by a similar concern, the International Society of Amyloidosis has created a nomenclature committee whose periodical reports try to keep pace with the ever-increasing knowledge of amyloid disorders in humans and animals (Westermark et al., 2007; Benson et al., 2018). Recent efforts toward a controlled vocabulary were also taken in the context of phase separation in living cells (Alberti, 2017; Shin and Brangwynne, 2017; Boeynaems et al., 2018), or while establishing guidelines to the use of Th-T in the presence of non-amyloid species (Gade Malmos et al., 2017). We now propose an ontological roadmap (Figure 2) and a systematized terminology (Table 1), considering the established nomenclature and the fundamental concepts of physical-chemical equilibrium. We depart from the definition of phase as a region in space with uniform density and composition at a given pressure and temperature (Prausnitz et al., 1998). Phase separation, therefore, refers to a change in density and/or composition that culminates in the formation of a new phase. The transition of a molecule *i* from phase *A* to the new phase *B* is driven by the difference in chemical potential $\Delta \,\mu =\mu_{\text{i}}^{B}-\mu_{i}^{A}$ that results from the different values of mole fraction *x*_i_ and activity coefficient γ_i_ within the two phases. Recall that

(1) $$\mu_{i}=\mu_{i}^{0}+R\,T\,\text{ln}\,\left(x_{i}\,\gamma_{i}\right)$$

**TABLE 1:** Glossary of terms used in protein aggregation phenomena.

Amyloid fibril	Insoluble, protease resistant aggregate with characteristic (i) fibrillar electron microscopic appearance, (ii) X-ray fiber diffraction pattern and (iii) histological staining reactions, including Th-T fluorescence and green birefringence in the presence of Congo red.
Amyloidosis	Any disease associated with the formation of amyloid deposits, i.e., deposits that have amyloid fibrils as main constituent.
Condensate	The denser phase formed upon phase separation.
Embryo	A metastable cluster formed as the result of random molecular collisions; embryos will tend to disintegrate if smaller than the critical nucleus size.
Liquid demixing	Separation of a solution into two coexisting liquid phases (compare with phase separation); also known as “liquid-liquid demixing.”
Mature amyloid fibril	A long, fully stable amyloid fibril consisting of intertwined protofilament units producing a wound structure that is resistant to 2% (w/v) sodium dodecyl sulfate (SDS) treatment.
Nucleation	The stochastic process by which nuclei are formed; primary nucleation occurs in the homogeneous bulk solution; secondary nucleation occurs on the surface of an already existing aggregate; heterogeneous nucleation occurs on the surface of a foreign substance.
Nucleus	A cluster formed as the result of random molecular collisions and with the ability to elongate into a fibril (compare with embryo).
Off-pathway oligomer	Stable oligomer that coexists with amyloid fibrils but is not an amyloid percursor; off-pathway oligomerization and amyloid fibrillation are competitive processes.
Oligomer	Multimeric species lacking the morphology and properties of amyloid fibrils.
On-pathway oligomer	A precursor of amyloid fibrils; the addition of pre-formed on-pathway oligomers accelerates the formation of amyloid fibrils without affecting the total amyloid conversion.
Phase separation	The formation of a new region with uniform density and composition; it can occur instantaneously by spinodal decomposition, or via the energy-activated process of nucleation.
Protofibril	Beaded chain with ∼5 nm diameter and <150 nm length that matures into amyloid fibrils.
Protofilament	Subunit of amyloid fibrils; smaller protofilaments with ∼1 nm diameter entwine to form larger protofilament units with 2.5–3.5 nm diameter.
Seed	Preformed amyloid particle able to accelerate the assembly of amyloid fibrils.

with $\mu_{\text{i}}^{0}$ being the standard chemical potential and *R* the universal gas constant. The depletion of phase *A* (and the concomitant enrichment of phase *B*) in component *i* proceeds until the equilibrium condition $\mu_{\text{i}}^{A}=\mu_{i}^{B}$ is verified, so that

(2) $$x_{i}^{A}\,\gamma_{i}^{A}=x_{i}^{B}\,\gamma_{i}^{B}$$

The formation of a new phase can occur instantaneously by spinodal decomposition, or via the energy-activated process of nucleation. In the latter case, thermal and compositional fluctuations result in the generation of embryos of phase *B* with non-zero surface free energy in the phase boundary. The thermodynamically unstable embryos tend to disintegrate back to phase *A*, unless they are larger than the critical size of the primary nucleus (Vekilov, 2010; Falahati and Haji-Akbari, 2019). Once this free energy barrier is overcome, phase transition will proceed at a faster rate if catalyzed by the secondary steps of growth (or elongation) and templated nucleation (or secondary nucleation) (Padrick and Miranker, 2002; Crespo et al., 2012; Meisl et al., 2016; Chatani and Yamamoto, 2018). Alternatively, the initially homogeneous phase *A* can be further destabilized by increasing *x*_i_ and/or by decreasing *T* to a point of spinodal decomposition where phase separation occurs by spontaneous amplification of infinitesimal phase fluctuations (Falahati and Haji-Akbari, 2019). The absence of a visible lag phase in aggregation progress curves does not necessarily mean that the critical nucleus has reached the minimum size of 1 molecule required for spinodal decomposition (Vekilov, 2010). Instead, it may happen that the presence of a nucleation barrier is concealed by (comparatively) fast elongation and secondary nucleation steps, thus originating the hyperbolic curves typical of downhill polymerization (Hurshman et al., 2004; Crespo et al., 2012).

As discussed above, the occurrence of kinetically trapped species is one of the obstacles for reproducible reporting of aggregation experiments. While corresponding to an unstable, high-energy state, the short-lived nucleation embryos are not included in the catalog of such intermediates. Primary nuclei, on the contrary, may have a fixed, well-ordered structure at the moment of their formation, or pass through local spatial ordering and structural annealing until a free energy minimum is eventually reached (Murray et al., 2017; Boeynaems et al., 2018). The sizes of primary and secondary nuclei can be estimated from the concentration dependences of kinetic parameters such as the duration of the lag phase and the limit aggregation rates (Cohen et al., 2013; Dovidchenko et al., 2014; Silva et al., 2018), but also through direct measurements using dynamic light scattering (Walters and Murphy, 2011; Silva et al., 2017) and small-angle X-ray scattering techniques (Vestergaard et al., 2007). In the hierarchy of amyloid fibril assembly, protofilaments are the primordial insoluble species and the fibrillar subunit of the β-sheet stacking (Teplow, 1998; Rochet and Lansbury, 2000; Khurana et al., 2003; Makin and Serpell, 2005). Protofilaments intertwine to form small and flexible protofibrils, which are classified as worm-like, rod-like or fuzzy, according to the morphological features displayed in, e.g., EM micrographs (Gosal et al., 2005; Gade Malmos et al., 2017). The elongation and intertwining of protofibrils give rise to typically straight and SDS-resistant mature fibrils with length often exceeding 1 μm and diameter around 10–20 nm (Rochet and Lansbury, 2000; Eisenberg and Sawaya, 2017; Gade Malmos et al., 2017). Instead of mature fibrils, the end product of amyloid assembly may consist of protofibrils, as in the cases of the L55P mutant of transthyretin at physiological (instead of acidic) pH (Lashuel et al., 1999), and of ataxin-3 containing a non-expanded (fewer than ca. 30 glutamine residues) polyglutamine tract (Carvalho et al., 2018). Equally, dense liquid droplets nucleated after local enrichment of aggregation-prone proteins can either mediate new disorder-to-order transitions during crystallization and amyloid fibril formation (Vorontsova et al., 2015; Aguzzi and Altmeyer, 2016), or develop into gel-like states with reduced fluidity and protein movement (Aguzzi and Altmeyer, 2016; Banani et al., 2017; Boeynaems et al., 2018). Pathway 1 in Figure 2 illustrates the formation of mature fibrils preceded by the occurrence of several intermediate states, including liquid droplets, gels, and protofibrils. Pathways 2 and 2′ show the possible coexistence of kinetically arrested states that, in this illustrative example, correspond to protofibrils and liquid droplets, respectively. Pathway 3 corresponds to the direct formation of protein crystals from globular protein.

Protein unfolding and oligomerization (represented in Figure 2 by green dashed lines) involve the interconversion between native, partially unfolded, denatured and oligomeric states of the protein. The equilibrium composition of these structurally distinct entities results, as in phase separation, from the balance of chemical potentials; however, each state of the protein is now characterized by different values of the standard chemical potential $\mu_{\text{i}}^{0}$ (Eq. 1) (Tanford, 1970). Although occurring within the same liquid phase, structural transitions might trigger subsequent phase separations as in the general case of protein misfolding diseases in which pathologic protein aggregation arises from the failure of a specific protein or peptide to adopt its native conformation (Chiti and Dobson, 2006). Also, insulin amyloid fibril formation is preceded by the aggregation of a precursor helical oligomer that later becomes the repeating unit of mature fibrils (Vestergaard et al., 2007), whereas several of the key proteins present in membraneless organelles have oligomerization domains that drive liquid demixing through multiplicative sticky interactions (Boeynaems et al., 2018). In a different example, amyloid fibrillation of non-expanded ataxin-3 decreases the concentration of soluble monomers, thus causing the dissociation of off-pathway oligomers to monomeric species (Silva et al., 2017). Because the different states of protein aggregation are in dynamic equilibrium, a possible rescue mechanism includes the sequestration of harmful species into less toxic aggregates or in phase separated compartments of the cell (Gosal et al., 2005; Haass and Selkoe, 2007; Boeynaems et al., 2018). Illustrating this protection mechanism, amyloid plaques isolated from AD cortex do not elicit toxic effects in rodent hippocampus unless they are solubilized to release toxic Aβ dimers (Shankar et al., 2008).

Systematizing protein folding intermediates and oligomeric species into organized categories is a challenging task due to the heterogeneity and elusive nature of both populations. Moreover, many proteins naturally occur as an ensemble of more than one polypeptide chain folded into a characteristic oligomeric conformation (Doyle et al., 2013), while others, known as intrinsically disordered proteins, can sample a continuum of conformations (Jarosz and Khurana, 2017; Darling et al., 2018). Folding intermediates have been detected for a limited number of proteins by measuring the exchange rates of hydrogen atoms between the main-chain amides and water (Englander et al., 2016), or by combining ^1^H liquid-state NMR and multivariate analysis (Malmendal et al., 2010). Figure 2 necessarily simplifies the folding landscape that is associated with pathogenic protein aggregation as different protein-protein interactions are required to maintain proteostasis in living cells. For example, the correct folding of prion protein (PrP) is assured by chaperones of the endoplasmic reticulum such as calnexin (Wang et al., 2010) and the proline cis/trans isomerase cyclophilin B (Ben-Gedalya et al., 2015). Conformational variations between the normal (PrP^C^) and the infectious (PrP^SC^) isoforms are responsible for the recruitment of PrP^C^ by PrP^SC^ during amyloid polymerization (Prusiner, 2001). The self-templating properties of PrP^SC^ are the basis for ultrasensitive tests for prion infections in biological fluids and tissues (Saborio et al., 2001; Orru et al., 2012). Likewise, the amplification of seeding-competent aggregates of Aβ peptide (Salvadores et al., 2014), α-synuclein (Fairfoul et al., 2016) and tau (Saijo et al., 2017) is a promising technological principle for the diagnosis of AD, PD, and tauopathies, respectively (Soto and Pritzkow, 2018). Amyloid seeding capacity can be used to evaluate whether a given intermediate is on-pathway or off-pathway to form amyloid fibrils. The maximal seeding potency is achieved upon the addition of pre-formed, sonicated fibrils. Conversely, off-pathway oligomers (Farmer et al., 2017; Hasecke et al., 2018), fibril polymorphs (Kodali and Wetzel, 2007; Falcon et al., 2015; Cao et al., 2019), and seeds pre-treated with aggregation inhibitors (Arimon et al., 2008; Oskarsson et al., 2018; Saelices et al., 2018) are expected to have lower seeding potencies. Amyloid fibrils produced from distinct proteins can be tested for conformational complementarity in cross-seeding experiments, also known as heterologous or heterogeneous seeding (Harper and Peterand Lansbury, 1997; O’Nuallain et al., 2004; Soto and Pritzkow, 2018).

## Methods to Experimentally Evaluate General Aggregation and Amyloid Formation

Significant experimental evidence must be gathered by complementary biophysical methods to determine if a protein or peptide does aggregate and if it is specifically able to form amyloid assemblies. Several computational approaches have been developed to predict aggregation-prone regions in (poly)peptide sequences (Belli et al., 2011; Pallarès and Ventura, 2019), but often experimental validation of aggregation propensity represents a considerable bottleneck (Grishin et al., 2020). Protein self-assembly is a complex process that might result in the formation of amorphous aggregates, different oligomers or amyloid fibrils, heterogeneous species that often coexist during the aggregation process. In this section, the methodological approaches required to identify general protein aggregation (see section “Monitoring the Presence of Protein Aggregates”) and to distinguish amyloid fibril formation specifically (see sections “Methods to Monitor the Conformational Properties of Amyloid Aggregates,” “Morphology of Amyloid Fibrils,” and “Fluorescence Techniques and Tinctorial Properties of Amyloids”) are summarized. In particular, we focus on the study of amyloid fibril formation, which can be addressed from the structural (see section “Morphology of Amyloid Fibrils”) and the kinetic point of view (see section “Fluorescence Techniques and Tinctorial Properties of Amyloids”). A minimum set of experiments is suggested to satisfy the criteria that generally define amyloid aggregates according to their conformational, morphological and tinctorial properties.

### Monitoring the Presence of Protein Aggregates

The formation of protein aggregates can be detected by a collection of orthogonal methods that report on different properties of these macromolecular assemblies.

#### Light Scattering and Turbidimetry

The aggregated states of proteins or peptides scatter the light passing through the aggregate solution proportionally to the size of aggregated particles. Therefore, the course of aggregation can be monitored by the measurement of an attenuation of incident light beam (turbidimetry) or by integration of an angle-specific scattering [static light scattering, SLS (Zhao et al., 2016)]. Turbidimetry is often taken as a linear descriptor of the kinetics of aggregation reactions; however, caution should be taken when comparing different proteins or conditions, since light is scattered as a function of both aggregate size and shape. SLS is able to determine the relative size of an aggregate by measuring the time-averaged intensity of scattered light, usually employing the same wavelength for excitation and detection at an angle of 90°. It reports on the molar mass (weight-average) and concentration of the aggregate.

#### Dynamic Light Scattering

Dynamic light scattering (DLS), also known as photon correlation spectroscopy or quasi-elastic light scattering, is a spectroscopy technique that measures the fluctuation of intensity of scattered light with time and is routinely applied to detect protein aggregates, as well as other nanoparticles (Zaccai et al., 2017).

Dynamic light scattering allows to derive two basic characteristics from a protein population. First, the mean hydrodynamic size, assuming a spherical geometry of the particle, and second, the polydispersity index of the solution. DLS is highly sensitive to the presence of aggregates because light scattering intensity scales with the second power of the mass of the light scattering particle. Therefore, low amounts of protein aggregates can be detected when the hydrodynamic radii are large enough. When the sample contains both monomeric and aggregated species, the monomer can only be adequately detected when the polydispersity index is low and the protein concentration is high. In this case, the particle size distribution plot shows multiple peaks indicative of a multimodal distribution.

#### Size Exclusion Chromatography

Size-exclusion chromatography (SEC) offers the possibility to identify, collect and determine the relative molecular weight of the different assemblies in an aggregated sample (Striegel et al., 2009; Burgess, 2018). Extra care must be exerted when dealing with labile oligomers. These can be potentially disrupted during the fractionation process, shifting the equilibrium between species as a function of the protein concentration. Moreover, large insoluble aggregates should be filtered out before analysis to prevent column clogging.

Size-exclusion chromatography becomes a versatile technique when coupled to multiangle light scattering (MALS) and differential refractive index (dRI) detectors, which allow to determine protein concentration, molecular weight (MW), size and conformation (Folta-Stogniew, 2006). The SEC-MALS instrument is calibrated independently of the column and does not depend on commercial reference standards, becoming the default method for the estimation of native MW on heterogeneous samples (Sahin and Roberts, 2012).

#### Intrinsic Fluorescence Spectroscopy

The sensitivity of the intrinsic fluorescence signal of tyrosine and tryptophan residues to their local environment has been extensively used to study protein conformation by fluorescence spectroscopy. A steady-state fluorescence spectrum is obtained by recording the emission fluorescence intensity of a sample excited at a fixed wavelength. In proteins, tryptophan fluorescence is owed to the side-chain indole group, displaying an absorption maximum at 280 nm and a fluorescence peak that is solvatochromic, ranging from 300 to 350 nm. Depending on the polarity of the local environment, the fluorescence emission maximum ranges from ∼308 nm for a tryptophan fully embedded in a hydrophobic pocket to ∼348–352 nm for a tryptophan fully exposed to solvent (Ghisaidoobe and Chung, 2014). Thus, the fluorescence of tryptophan in monomeric and aggregated states of proteins is significantly different (Bobone and van de Weert, 2014). The fluorescence of tyrosine is due to its side-chain phenolic ring, with excitation and emission maxima at 260 nm and 305 nm, respectively. When compared to tryptophan, the fluorescence signal of tyrosine is less sensitive to environmental changes, as a consequence of the lack of dipole reorientation in the excited phenol. Still, since tyrosine is on average three times more abundant than tryptophan in polypeptides, its signal also differs significantly between the aggregated and soluble states of proteins.

### Methods to Monitor the Conformational Properties of Amyloid Aggregates

Although the nature and self-assembly mechanisms of protein amyloids differ, most of them share a final fibrillar morphology highly enriched in intermolecular cross-β structure, whose presence can be confirmed by different biophysical methods.

#### Circular Dichroism Spectroscopy

Circular dichroism (CD) relies on the differential absorption of left and right circularly polarized light. Optically active chiral molecules and chemical groups absorb preferentially in one direction of the light. Thus, peptide bonds, aromatic amino acid side chains and disulfide bonds in proteins act as conformation reporters, providing information about the secondary structure composition (α-helix, β-sheet, disorder). In the far UV region of the spectrum (190–260 nm) a maximum at 196 nm and a single minimum at 218 nm is attributed to a β-sheet conformation; meanwhile, a maximum at 192 nm and two minima at 208 nm and 222 nm are indicative of α-helical content, whereas a minimum at 198 nm correlates with the presence of disordered regions (Cristovao et al., 2019). The shift from an initial native conformation to an amyloid β-sheet structure often exacerbates the minimum at 218 nm (Kelly et al., 2005; Vadukul et al., 2019). The presence of additional molecules in the sample, such as reducing agents, organic molecules and excipients, should be taken into account since they might interfere with the measurement, precluding detection of the expected transition.

The far UV spectra of aggregated samples are usually complex and require the use of deconvolution algorithms (SELCON, VARSLC, CDSSTR, K2d2, K2d3, BeStSel, DICHROWEB server) (Provencher and Glockner, 1981; Manavalan and JohnsonJr., 1987; Andrade et al., 1993; Sreerama and Woody, 2000; Whitmore and Wallace, 2004; Micsonai et al., 2015) to quantify the contribution of the different secondary structure elements to the signal. However, care should be taken with these estimates, even in the analysis of the variation of secondary structure content, which should be favored over absolute determinations. The presence of aggregates often leads to distortions in the CD spectra (differential absorption flattening) that might lead to errors in the estimation of secondary structure content, as most algorithms are optimized for soluble proteins. Of note, one algorithm – BeStSel – is especially suited for secondary structure determination of proteins with high β-sheet content, such as those found in amyloid protein aggregates (Micsonai et al., 2015).

The CD spectrum in the near UV region (260–320 nm) arises from the contribution from aromatic amino acids and provides information on the tertiary structure, complementary to that gathered from intrinsic fluorescence measurements.

#### Fourier-Transform Infrared Spectroscopy

Fourier transform infrared (FTIR) spectroscopy is especially suitable to determine the existence of β-sheet secondary structures in aggregated samples. The presence of a band at 1620–1640 cm^–1^ in the amide I region of the infrared spectrum is a signature of β-strands. Intramolecular (native β-sheet) signals fall typically in the 1630–1640 cm^–1^ range, whereas the intermolecular β-sheets characteristic of aggregates usually peak at 1620–1630 cm^–1^ or even lower wavenumbers (Hiramatsu and Kitagawa, 2005).

The FTIR spectrum is particularly sensitive to the presence of additives in the sample, such as TFA, DMSO and reducing agents; therefore, the signal of the buffer should be subtracted from that of the sample. Because the vibration signal of water maps in the amide I region (1630 cm^–1^), the FTIR spectrum must be obtained by drying out the sample to an hydrated protein film when using Attenuated Total Reflection sampling devices or by exchanging H_2_O with D_2_O for transmission experiments when using DTGS (deuterated triglycine sulfate) and MCT (mercuric cadmium telluride) detectors (Cristovao et al., 2019).

The peaks in the FTIR spectrum are identified by curve fitting using Fourier self-deconvolution, or/and by analysis of the inflection points in the second derivative of the spectrum curve. Fitting of the FTIR spectrum does not provide a unique solution and thus the fitting parameters become critical: a minimum number of peaks should be used, the maxima of the curve-fitted peaks should correspond to the evident maxima in the raw data, and, generally, curve-fitted peaks should present similar full-width and half-height values. This technique can be used to study and compare protein aggregates obtained *in vivo* and *in vitro* (Shivu et al., 2013). Traditional FTIR analysis only reports on bulk composition and even FTIR microspectrometers, despite their extensive applications [e.g., in cell biology (Bouyanfif et al., 2018)], only provide resolution down to 2–5 μm, which is insufficient for the analysis of individual fibrils. However, by combining IR with atomic force microscopy in Infrared Nanospectroscopy it is possible to acquire both morphological, nanomechanical and nanoscale-level (10–20 nm) chemical IR absorption spectra and maps from protein aggregates, single cells and liquid-liquid phase condensates (Ruggeri et al., 2020). This approach is particularly useful in analyzing complex mixtures with co-existing amyloid, amorphous aggregates and pre-fibrillar aggregates with different secondary structure (Ruggeri et al., 2015, 2016; Galante et al., 2016; Waeytens et al., 2020).

#### Proteinase K and Mixture of Proteases Digestion

Complementary to the techniques mentioned above, the relative resistance of amyloid fibrils to proteinase K digestion is commonly used for their characterization, especially in the case of prionic proteins. Proteinase K is a serine protease that cleaves peptide bonds on the carboxy-terminal side of aromatic and aliphatic amino acids. Despite the high processivity of proteinase K in disordered and regular secondary structures, its proteolytic activity is rather limited in cross-β-sheet structures, which are characteristic of amyloid fibrils (Kushnirov et al., 2020). The identification of proteinase K-resistant amyloid cores can be achieved by two main approaches: adding different proteinase K concentrations at the end-times of aggregation reactions or following the time-course of the digestion at a given proteinase K concentration. The results of the digestion experiments can be monitored with several techniques, such as SDS-PAGE and protein immunoblotting, mass spectrometry, or electron microscopy. In some cases, a mixture of proteases can be used instead of proteinase K to identify the core of amyloid fibrils (Selivanova et al., 2016; Surin et al., 2020).

#### X-Ray Diffraction and Scattering Techniques

X-ray diffraction and small-angle scattering techniques, including small-angle X-ray scattering (SAXS) and small-angle neutron scattering (SANS) are considered gold standard techniques for the analysis of the structural properties of amyloid fibrils.

The X-ray fiber diffraction pattern of partially aligned amyloid fibrils displays a characteristic arrangement with a meridional reflection (vertical axis) at 4.7–4.8 Å and an equatorial reflection (horizontal axis) at 10–12 Å (Figure 1B), which report on the distances between β-strands that are associated via hydrogen-bonding perpendicular to the fibril axis and between the adjacent β-sheets running parallel to the axis, respectively. The meridional signal is generally very sharp and intense due to the repeating nature of the β-strands along the fiber axis. The equatorial signal is generally weaker and more diffuse and its position can vary depending on the amino acid composition of a constituent peptide (e.g., large aromatic side chains will result in a larger sheet spacing). This is the reason why the common amyloid fold is known as cross-β (Morris and Serpell, 2012). Low angle signals are often also observed, which arise from chain length and/or protofilament packing.

Small-angle scattering techniques are one of the best-suited biophysical approaches to study the conformational properties of the different oligomeric forms appearing along an amyloidogenic process and how they evolve into mature fibrils. In theory, one can decompose the time-resolved SAXS and SANS data into the scattering intensity profiles of the individual forms (structural information), along with their relative populations (kinetic information), without the need to isolate the transient or co-existing species (Langkilde et al., 2015). Nonetheless, the use of this technique is limited to specific cases because it requires highly concentrated and stable samples, containing only a few oligomeric species simultaneously.

#### Sedimentation Techniques: Separation and Size Distribution

While complex mixtures of aggregated protein can be imaged by a variety of different techniques (see section “Morphology of Amyloid Fibrils”), separation of insoluble species is not straightforward. An increase in particle mass during protein aggregation can be exploited for detection, isolation and characterization of both high-molecular weight intermediates and final filaments. Several ultracentrifugation protocols have been adopted for analyzing protein aggregates in cellular and animal models of various human proteinopathies including prion protein amyloidosis (Vey et al., 1996), a familiar tauopathy (Berger et al., 2007) or sporadic AD (Skrabana et al., 2017). The ability to differentially isolate protein conformers with distinct physical properties by sedimentation at high speed can be used also in *in vitro* experiments of protein aggregation. Insoluble fibrils and larger aggregates pellet quickly around 14,000–16,000 × *g* in aqueous buffers, but smaller species such as oligomers or shorter fibril fragments sediment more slowly even under high-speed centrifugation above 50,000 × *g* (Mok and Howlett, 2006). It is sometimes possible to separate different fibrils based on their morphology. Straight fibrils of human apolipoprotein ApoC-II, which dominate in the presence of phospholipid micelles, sediment more rapidly than flexible ribbons formed in the absence of lipids, making it possible to pellet the two populations at 14,500 × *g* and 350,000 × *g*, respectively (Mok et al., 2015). Similar approaches have separated linear versus closed-loop fibrils of ApoC-II (Yang et al., 2012), while complex mixtures of huntingtin aggregates have more simply been fractionated by pelleting a mixture of inclusions, soluble but large oligomers and cell debris from lysed cells (14,000 × *g* for 10 min) followed by centrifugation of the resuspended pellet through a desalting column, which allows separating the soluble oligomers from the insoluble material (Ormsby et al., 2013). Just as importantly, if the relationship between the molecular weight of fibrillar species and their diffusion coefficient is known (MacRaild et al., 2003), it is possible to estimate the size distribution of fibrils using sedimentation velocity analysis (Yang et al., 2012), which measures how quickly fibrils move through a centrifugal field. It is even possible to selectively monitor fibril sizes in the presence of non-fibrillar components (e.g., chaperones) using fluorescence detection and fluorophore-labeled fibrils; this allows to assess changes in the size distribution and lateral association of a given protein (Binger et al., 2013). As an alternative approach, OptiPrep gradients have been used to isolate and characterize protein aggregates associating with membrane-like domains (Vey et al., 1996).

### Morphology of Amyloid Fibrils

Once the β-sheet content has been confirmed by the methods above, the morphology of the amyloid fibrils should be examined by imaging techniques.

#### Transmission Electron Microscopy

The visualization of transmission electron microscope (TEM) images at high resolution provides information on the morphology, homogeneity and size of the amyloid fibrils. In order to obtain high-quality TEM data, samples deposited on carbon-coated copper grids should be negatively stained with a heavy metal solution [typically 2% (w/v) uranyl acetate solution in water] to increase contrast. It should, however, be taken into account that TEM images may display structural artifacts due to dehydration and staining during the sample preparation process (Gras et al., 2011).

#### Atomic Force Microscopy

Atomic force microscopy (AFM) allows direct visualization of amyloid fibrils in aqueous solutions, providing information about their structural and mechanical properties under physiological-like conditions (Ruggeri et al., 2019). AFM images have nanometer-resolution, allowing to assess the fibril contour length, width, height and periodicity or the high-order assembly of single protofilaments into mature fibrils (Adamcik et al., 2012). These properties can be measured at final time-points or directly during the assembly reaction. Besides, it is possible to define the packing scheme and polymorphic state of the amyloid fibrils (e.g., twisted, helical ribbon) by measuring the height profile of fibrils along their contour length and the shape of height profile. An advantage of AFM, when compared to TEM, is that it allows measuring forces and elastic properties of amyloid assemblies with piconewton resolution.

Several additional methods, such as solid-state NMR and cryo-EM, provide valuable information about the morphology and structural features of amyloid fibrils at high resolution. These methods and their applications to uncover the atomic structures of amyloid fibrils have been extensively covered in recent reviews (Iadanza et al., 2018a; Fitzpatrick and Saibil, 2019). Although key to explore correlations between structure and toxicity, they need advanced equipment and go beyond a *minimum requirement* for determining that a polypeptide assembles into amyloid fibrils.

### Fluorescence Techniques and Tinctorial Properties of Amyloids

It is well established that regular fibrillar structures have the ability to bind small molecules at their surfaces and cavities with a concomitant alteration of the optical properties of the binding compounds. Th-T and CR are two of these molecules, and have been extensively used for the detection of amyloid structure in fibrils and deposits.

#### Thioflavines

The Th-T assay measures the increase in emission fluorescence signal of Th-T as amyloid fibrils grow. The enhanced fluorescence can be detected by spectroscopy or visualized by epifluorescence or confocal microscopy. In all techniques, the molecule is excited at 445 nm and emission fluorescence is recorded in the 470–500 nm range, usually with a maximum around 482 nm (Biancalana and Koide, 2010).

Th-T fluorescence enhancement is not a quantitative parameter since it is strongly dependent on the fibril morphology. In some cases, amyloid fibrils may be present, but do not display fluorescence because the rotation movement of the Th-T molecule is not sufficiently impeded. On these occasions, Th-T fluorescence anisotropy provides an alternative technique for the study of amyloid aggregation (Sabate and Saupe, 2007). Also, the maximum excitation and emission wavelengths may change slightly depending on each particular amyloid structure (Sabate et al., 2013).

Th-S is a mixture of compounds that results from the methylation of dehydrothiotoluidine with sulfonic acid. As such, its molar concentration cannot be accurately calculated, which in addition to its high fluorescence background make this dye sub-optimal for spectroscopic measurements *in vitro* (Espargaro et al., 2012). However, Th-S presents the advantage of being able to permeate through cell membranes, thus allowing to detect intracellular amyloid aggregates, even in living cells. Accordingly, it has been vastly used in the histological staining of amyloids and to image purified amyloid material (Figure 1C).

#### Congo Red

The CR dye has been broadly used to detect amyloid aggregates in tissues and *in vitro*. The absorption spectra of CR alone and in the presence of amyloids are recorded in the visible region of the light spectrum (300–700 nm) and compared (Yakupova et al., 2019). The binding of CR to amyloids induces a spectral red shift, with maximum absorbance change occurring around 540 nm. As in the case of Th-T assays, the CR spectrophotometric assay is not quantitative.

Birefringence originates from the decomposition of a ray of light into two rays when it passes through certain anisotropic materials, such as crystals. The fixation of CR molecules along the axis of the amyloid fibrils usually causes apple-green birefringence when viewed through cross-polarized light, providing an assessment of the amyloid nature of protein aggregates complementary to CR absorbance measurements.

#### Other Amyloid-Staining Molecules

The use of Th-T and CR for amyloid detection has limitations. Many non-amyloid molecules can also exhibit birefringence under cross-polarized light in the presence of CR, such as phosphate salts, urea, and other types of fibers like hair. Moreover, CR is a pH indicator and, accordingly, its absorbance spectrum is strongly dependent on the solution pH, being useless under acidic conditions. The absorption and emission spectra of some molecules, such as flavins and reduced NAD(P)H overlap with that of Th-T. Also, some polyphenolic anti-aggregational compounds, such as curcumin or epigallocatechin, exhibit fluorescent properties similar to those of Th-T (Hudson et al., 2009). In a similar way, the auto-fluorescence generated by some aldehyde compounds used as tissue fixatives significantly interferes with the histological detection of amyloids using this dye. In light of these problems (Viegas et al., 2007), novel fluorescent dyes with improved sensitivity and specificity properties have been developed.

As Th-T, the ProteoStat dye is a rotor molecule that intercalates into the cross-β structure, leading to a strong red fluorescence with excitation and emission maxima at 500 nm and 600 nm, respectively (Shen et al., 2011; Navarro and Ventura, 2014). Compared to Th-T, ProteoStat staining produces a stronger fluorescent signal with a wider linear dynamic range in a broad range of pH values (4–10) and avoids spectral overlap with the autofluorescence signal of membranes and co-factors. ProteoStat is particularly indicated if working in the presence of RNA, when the use of Th-T is unsuited, because it produces huge distortions of the baseline (Sugimoto et al., 2015; Liu et al., 2017).

Heptamer-formyl thiophene acetic acid (hFTAA) is yet another amyloid detection dye. hFTAA exhibits distinct shifts in its emission spectra when bound to different amyloid species and polymorphs, finding application in the differentiation of amyloid (sub)types *in vivo* and in monitoring changes of amyloid structure and composition over time (Klingstedt et al., 2011, 2013; Sjolander et al., 2016).

Molecules such as 1-anilinonaphthalene-8-sulfonate (ANS) and its dimeric analog 4,4′-bis-1-anilinonaphthalene-8-sulfonate (Bis-ANS) are barely fluorescent in polar solvent, but become highly fluorescent in an apolar environment. In the presence of aggregates a blue shift and an increase of their emission maxima occur, due to their binding to the hydrophobic clusters exposed in such assemblies. ANS and bis-ANS are particularly useful in the detection of the low concentrations of protein aggregates populating the early stages of the reaction (de Groot et al., 2007).

A Th-T derivative, called Pittsburgh compound B (PiB) is able to enter the brain and is used as a radioactive tracer for *in vivo* PET imaging of amyloid beta pathology in AD (Klunk et al., 2004).

## Guidelines for Reporting Protein Aggregation Experiments: The Mirragge Table

Given the diversity of oligomeric species and folding states, the choice of protein purification and handling procedures plays a crucial role in determining where in the aggregation map (Figure 2) are the departing and arrival points located, and by what pathways are they connected. Therefore, we propose a set of guidelines for presentation and publication of data related to polypeptide aggregation that ensure an adequate and accurate description of the experimental results. The relevant information that we propose to be reported is divided into two parts: sample preparation and quality and incubation conditions. Ideally, the methods section of each publication should contain the information briefly described in Table 2.

**TABLE 2:** Experimental details that should be reported for protein aggregation assays.

Reported parameter		Information that should be reported
Sample stock	*Sample source and storage conditions*	Description of protein source – commercial (supplier and reference), recombinant (including expression and purification), or other (e.g., extracted from tissue); description of how the protein or peptide stock was preserved (lyophilized or stored in solution); if stored in solution indicate storage buffer, sample concentration, storage temperature, freezing/thawing conditions, as well as material and supplier of vials used for sample storage.
	*Sample purity and concentration*	Indicate the methods (SDS-PAGE, HPLC, etc.) used to determine sample purity, as well as those used to determine the exact concentration of the stock sample in the assay. The purity of the sample stock as evaluated by SDS-PAGE should be indicated and higher than 95%. Indicate whether the sample was tested for the presence of “invisible” components such as nucleic acids.
	*Sample concentration*	Indicate the final concentration of the sample in the assay.
	*Sample preparation prior to assay*	Mention if the protein/peptide stocks were pre-treated, filtered or centrifuged; these procedures are recommended to remove pre-aggregated forms.
	*Assay buffer*	Indicate the composition, concentrations and pH value of the buffer or solvent stock.
	*Assay volume*	The final volume of the sample, as well as the volume of the tube/well in which the aggregation assay is performed should be reported since interfaces (liquid-solid and liquid-air) influence protein aggregation. The method used to prevent sample evaporation should also be mentioned when using small sample volumes.
	*Additives*	List ALL components that could be found in the aggregation assay (even those that may exist in minute amounts). For example, if an aggregation modulator is dissolved in DMSO, it is important that all samples (including control) contain the same final concentration of DMSO.
*Experimental procedures, type of equipment used and system-dependent parameters*	*Plate or vial*	Report the type of material and geometry of the vial or microplate well (binding versus non-binding surface, bottom versus top-reading, square versus round bottom, etc.) used in the assay. Include information on the microplate/vial material, including supplier and reference code.
	*Temperature*	Report the assay temperature. In particular, mention if samples/buffer are preincubated at the assay temperature: fluorescence drifts may be observed at the beginning of the experiment resulting from temperature shifts.
	*Agitation*	Indicate whether an orbital shaker, a thermomixer or magnetic stirring was used (describing shape, size and material of stirrers), the type of shaking (orbital, linear, etc.) and speed. If applicable, indicate if beads were included (material, size, number of beads). Measurement cycles and the pre-shaking agitation procedures should be clearly specified.
	*Time*	The total duration of the aggregation reaction should be reported.
	*Equipment for measuring aggregation kinetics and its settings*	Indicate the device make and model, control software, and general settings used (e.g., filter bandpass and bandwidth/monochromator settings).
	*Reporters*	Provide the exact amount of reporter (if used) employed for measurement and any pre-treatment of the sample.
	*Data analysis and raw data that should be preserved for publication*	Indicate the software (including version) used for image and data analysis and specify the equation applied for fitting kinetic data obtained from aggregation assays.

**FIGURE 3:**
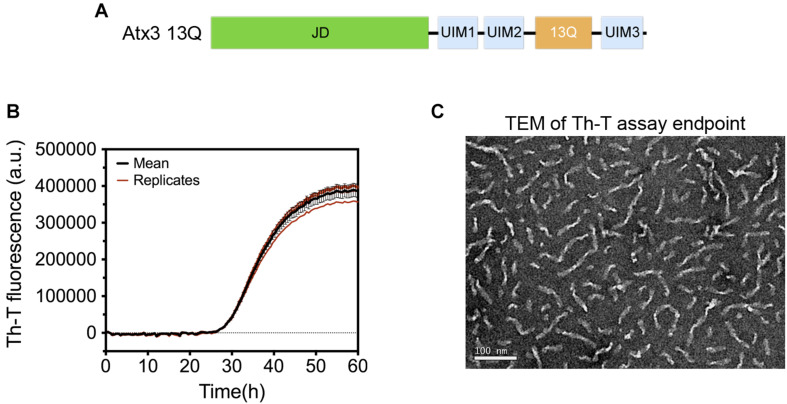
Aggregation of human ataxin-3 (UniProt accession code: P54252-2). **(A)** Schematic representation of the structural organization of ataxin-3, with the N-terminal globular Josephin domain (JD) and the flexible C-terminal tail encompassing the ubiquitin-interacting motifs (UIMs) and the polyQ tract containing 13 glutamine residues (13Q); **(B)** Representative graph of human ataxin-3 aggregation at 37°C monitored by Th-T fluorescence. **(C)** TEM image of ataxin-3 protofibrils at the end of the aggregation assay (∼60 h).

A pre-filled MIRRAGGE table using the aggregation of the protein ataxin-3 (Figure 3) (Gales et al., 2005; Almeida et al., 2015) and of a peptide from the Mot3 yeast prion (Sant’Anna et al., 2016; Fernandez et al., 2017) as examples is provided as Supplementary Information (Supplementary File MIRRAGGE_template.xls) illustrating the minimum information that we suggest should be included to ensure reproducible (poly)peptide aggregation experiments. A large part of the minimum information required in the MIRRAGGE table concerns the sample preparation steps that precede the aggregation assay itself. Starting by the unambiguous identification of the protein molecule, it is particularly important for curators of public databases that the UniProt accession number, the species of origin and, if applicable, the presence of affinity tags are provided for the molecules under investigation (Orchard et al., 2007; Trewhella et al., 2017). For synthetic peptides, the chemical nature of the N and C termini (whether capped or uncapped) should be made clear as well as the buffering salt (e.g., TFA). Besides the essential information about the sample source, namely the protein expression system, the purification and polishing protocols, or, in the case of samples obtained from commercial sources, the catalog number and the name and location of the supplier, the MIRRAGGE table can also encompass any additional information or experimental detail found to be important for the outcome of aggregation. This is acchieved by including a flexible field of “additional key information” at the end of the tabular descriptions of the purification protocol and of the aggregation assay, wherein relevant remarks concerning the aggregation state of the protein, sample collection procedures in gel filtration chromatography, the occurrence of contaminants or co-solvents, the procedure adopted for removal of air bubbles, a critical sequence of reagent addition, etc., can be emphasized. A comprehensive characterization of the aggregation assay in terms of the total volume of reaction, plate/cuvette/vial geometry and material, method of evaporation control, size and material of beads (if present), and type of agitation is required on account of the effects of interfaces and shear flow on protein aggregation (Giehm and Otzen, 2010; Bekard et al., 2011; Yoshimura et al., 2012; Ferreira et al., 2016; Koepf et al., 2018). As a major determinant of phase separation, protein concentration is discriminated (i) before storage of the purified protein, (ii) immediately before aggregation, e.g., after the final filtration step, and (iii) during aggregation. The concentration of protein or peptide is provided, together with the information about MW, extinction coefficient and the quantification method adopted. Freeze-thawing stress can lead to the cold denaturation of the protein, its adsorption at the ice-liquid interface with subsequent partial denaturation and aggregation, or to drastic pH changes in buffers like sodium phosphate or succinate (Hawe et al., 2009). It is recommended to control the quality of thawed samples to judge whether additional polishing steps are required before the aggregation assay. Cohen et al. (2013) reported two rounds of gel filtration post-thawing on purified Aβ42 peptide to ensure pure monomer at the beginning of the kinetic assay. In the case of ataxin-3, increased reproducibility is achieved by adding a re-polishing step immediately before the aggregation assay, separating the predominant monomer from contaminant oligomeric species putatively formed during the freezing-thawing process (Silva et al., 2017).

In conclusion, this review aims to provide an overview of the biophysical principles underlying protein self-assembly in its multiple shapes, and compiles detailed information on the experimental validation of polypeptide aggregation, with a focus on amyloid fibril formation. It results from several discussions on this topic in the context of the COST action BM1405 [Non-globular proteins – from sequence to structure, to applications in molecular physiopathology (NGP-net)]. We aim to endow both experienced researchers and newcomers to the field with a set of guidelines to enhance reproducibility in (poly)peptide aggregation experiments. The MIRRAGGE table template compiles a recommended reporting framework that is expected to provide a minimum set of information required for replicating protein aggregation experiments and swiftly discriminate protein assembly into amyloid fibrils from amorphous protein aggregation.

## Author Contributions

PMM and SN revised the literature and wrote the manuscript. AS, ZS, FF, and MFP designed the template MIRRAGGE table, with contributions from all authors. PJBP, FP, ZB, MB, OVG, ZG, CMG, AP, LCS, RS, VS, MZ, and DEO participated in the discussion of the minimum requirements for publication of experimental data on (poly)peptide aggregation, and contributed for the preparation of the list of experimental methods and details that should be reported for protein aggregation assays. SM-R and SV designed the review focus and finalized the manuscript. All authors contributed to the article and approved the submitted version.

## Conflict of Interest

The authors declare that the research was conducted in the absence of any commercial or financial relationships that could be construed as a potential conflict of interest.
